# Effect of acupuncture method of removing-stasisand resuscitating on the consciousness of patients with severe traumatic brain injury: A randomized clinical trial

**DOI:** 10.1097/MD.0000000000022056

**Published:** 2020-09-04

**Authors:** Jun-Jun Pan, Xiao-Zhou Hou, Ping Wang, Wei Li, Qing Zhang, Tao Dong

**Affiliations:** Wang Jing Hospital of China Academy of Chinese Medical Sciences, Beijing, China.

**Keywords:** acupuncture, clinical trials, protocol, severe traumatic brain injury

## Abstract

**Introduction:**

With the development of social economy, transportation and various infrastructures have also developed, but it has objectively increased the number of patients with head injury. Although the current craniocerebral medicine technology continues to advance, long-term bed rest and other complications have led to an insignificant decrease in the mortality rate of coma patients. It is not uncommon for patients with disturbance of consciousness caused by head injury in major hospitals.

**Methods/design:**

This will be a retrospective, single-blind clinical observational study. We will select 50 cases that meet the subject's selection criteria. According to whether they received acupuncture treatment or not, they will be randomly divided into 2 groups, namely treatment group and control group. The control group will be given conventional Western medicine treatment, and the treatment group will be given acupuncture method of removing-stasis and resuscitating treatment on the basis of the control group.

**Discussion:**

Our purpose is to observe the role of acupuncture method of removing-stasis and resuscitating in promoting the recovery of patients with severe head injury. We aim to provide more evidence-based medical evidence for acupuncture treatment of this disease.

**Trial registration:**

ClinicalTrials.gov, ChiCTR2000034732, Registered on 19 July 2020

## 1 Introduction

Craniocerebral injury is damage to the brain tissue caused by violence directly or indirectly acting on the head. According to the Glasgow Coma Scale (GCS) method, it is determined that a person who is in a coma for more than 6 hours or becomes unconscious again after injury is a severe head injury. Craniocerebral injury manifests as disturbance of consciousness, headache, nausea, vomiting, seizures, limb paralysis, sensory disturbance, aphasia, and hemianopia. Skull base fractures can cause cerebrospinal fluid otorrhea; brainstem injury can cause disturbances in consciousness and breathing and circulation. In severe cases, brain herniation can endanger life. Among them, the change of consciousness after head injury is 1 of the most common symptoms of neurosurgery patients with head injury. Direct or indirect violence acting on the head and causing brain tissue damage is the main reason for craniocerebral injury and consciousness disturbance. Wang Yuhu^[1]^ discussed the causes of the onset of coma patients and believed that various lesions inside and outside the brain and the ascending activation system of the brainstem reticular structure can lead to disturbance of consciousness. With the continuous improvement of medical technology, the mortality rate of head trauma has decreased, but the mortality rate of severe head injury is still between 30% and 50%.^[2]^ Although the current craniocerebral medicine technology continues to advance, long-term bed rest and other complications have led to an insignificant decrease in the mortality rate of coma patients. It is not uncommon for patients with disturbance of consciousness caused by head injury in major hospitals. Every year, about 2 million people in China are in a coma due to head injury, and about 200,000 of them are in a persistent vegetative state. Unconsciousness within a week of trauma can easily lead to complications and comorbidities and become an important cause of death. Therefore, early awakening and reducing complications and complications are the key to reducing mortality. A large number of patients with impaired consciousness only rely on drugs to promote awakening with little effect, and long-term large amounts of drugs are likely to bring many toxic side effects to patients. In addition, the continuous coma adds a huge psychological and economic burden to the family members of the patient, so the recovery of the disease is particularly urgent. Traditional Chinese Medicine (TCM) believes that blood stasis to block the brain collaterals is the basic pathogenesis of head injury. The treatment should be based on removing blood stasis and resuscitation. As an important part of TCM, acupuncture therapy is widely used in the treatment of this disease. There are many traditional acupuncture treatment methods, mainly including “Xing Nao Kai Qiao” acupuncture, electroacupuncture, scalp acupuncture and ear acupuncture. Among them, the “*Xing Nao Kai Qiao*” acupuncture method established by Shi Xuemin^[3]^ has a definite effect on the pathological factor of stroke with congestion. To further explore the effect of acupuncture method of removing-stasis and resuscitating on the consciousness of patients with severe traumatic brain injury, we designed this experiment. Our purpose is to observe the role of the method of removing-stasis and resuscitating in promoting the recovery of patients with severe head injury. The specific plan is reported as follows.

## 2 Methods/design

### 2.1 Study design and settings

**Figure 1:**
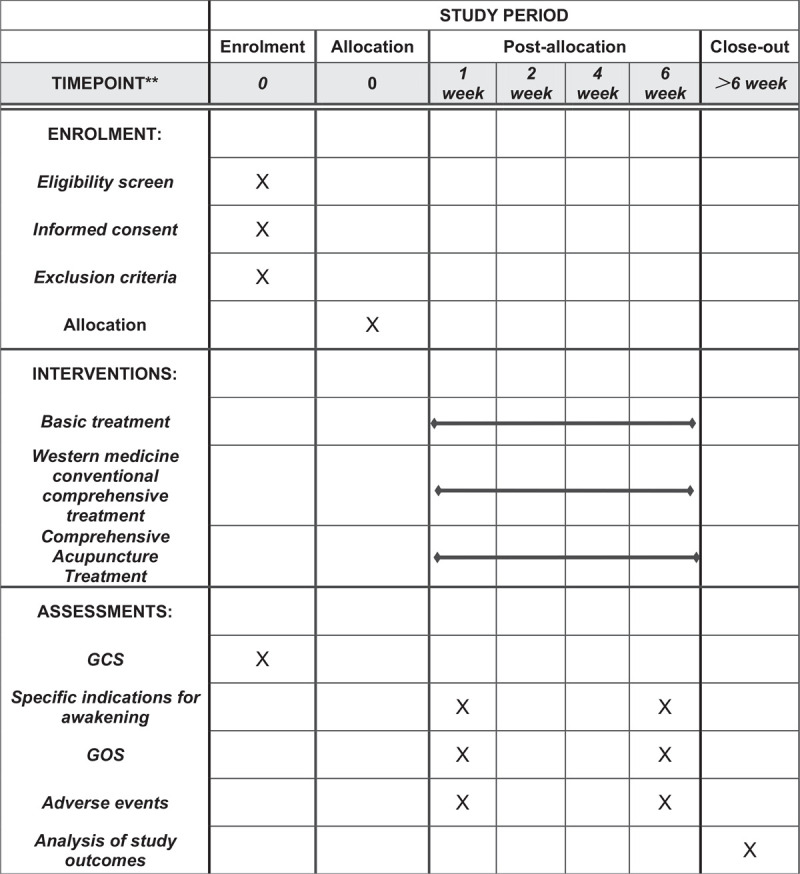
SPIRIT figure for the schedule of enrollment, interventions, and assessments. GCS = Glasgow coma scale, TCM = traditional Chinese medicine.

This will be a retrospective, single-blind clinical observational study. We will select 50 cases that meet the subject's selection criteria. According to whether they received acupuncture treatment or not, they will be randomly divided into 2 groups, namely treatment group and control group. The control group was given conventional Western medicine treatment, and the treatment group was given acupuncture method of removing-stasis and resuscitating treatment on the basis of the control group. After acupuncture, the needles are kept for half an hour, and acupuncture is performed every 10 minutes. Treatment is once a day, 5 times a week, with 2 days of rest, and 10 times is a course of treatment. Specialized researchers will record the unique clinical manifestations of the 2 groups of patients before and after treatment, coma GCS scores before and after treatment, recovery time, recovery rate, clinical efficacy and cure rate. We used statistical software SPSS25.0 to process the obtained data, and evaluated the clinical efficacy of the 2 groups based on the calculated results. (Fig. 1)

### 2.2 Participants and recruitment

#### 2.2.1 Participants

The case of this study will come from the Department of Neurosurgery of Wang Jing Hospital of China Academy of Chinese Medical Sciences, and the hospitalized patients who were diagnosed with craniocerebral injury and coma were diagnosed by Western medicine.

#### 2.2.2 Inclusion criteria

(1) Have a history of head trauma, and be diagnosed by head imaging examination;
(2) Age < 65 years old;
(3) In a coma 6 h after injury, GCS score <8;
(4) Those who have voluntarily signed the informed consent form;

#### 2.2.3 Exclusion criteria

(1) Pregnant women, formerly mentally ill patients and senile dementia;
(2) People with impaired consciousness caused by the primary disease;
(3) During observation and treatment, the patient has severe vital signs disorder, irreversible brain stem damage, respiratory and circulatory failure, and serious complications such as high fever and infection;
(4) Patients with tumor and other organ failure;

#### 2.2.4 Rejection, fall-off and suspension standards

(1) During the course of treatment and observation, the patient had other serious complications such as re-bleeding within a day and repeated intracranial hematoma removal;
(2) During the treatment process, the family members of the patient did not cooperate, voluntarily gave up the treatment, or quit the treatment voluntarily because of other circumstances;
(3) The cause of the coma cannot be determined, or the efficacy cannot be determined due to incomplete observation data;

### 2.3 Randomisation, allocation concealment and blinding

We will use the closed envelope method for random grouping. According to the order in which patients will be enrolled, the study objects will be numbered 1 to 50, and the random number and allocation group corresponding to the enrollment order will be obtained. Seal the distribution result in an opaque envelope, and put the serial number on the surface of the envelope. Random envelopes and assignments are in charge of special personnel who are not involved in treatment and evaluation. After baseline data collection, patients will be randomized before discharge. The assessor is responsible for delivering the resistance training intervention, in addition to outcome measure assessment at all time points; therefore, she will not be blinded to group allocation. It will also not be possible to blind participants to their respective group allocation.

### 2.4 Sample size determination

Since this is a pilot study, we will not perform calculations to determine the sample size for this study. Instead, the focus will be on the feasibility and safety of the research. We plan to recruit at least 60 participants with a retention rate of 80%.

### 2.5 Intervention method

#### 2.5.1 Basic treatment protocol

We will require patients to stay in bed absolutely and give them oxygen to keep their airways open. Patients with severe breathing disorders may undergo tracheotomy. Patients with indications for brain surgery will be treated with surgery. Our goal is to prevent and treat cerebral edema, gastrointestinal bleeding and other complications. In addition, we will also carry out routine neurosurgical treatment and nursing care for patients, such as infection control, supplementation of energy polarizing fluid and nutritional supply. When the patient's vital signs and condition are stable, they will be given acupuncture to promote awakening and assist in treatment.

#### 2.5.2 Control group

The control group will be given conventional comprehensive treatment of western medicine, dehydration, hemostasis, and nourishment of brain cells (intravenous infusion of monosialic acid ganglioglyceride 80 mg, 1 time/d, after 2 weeks of continuous use, change to 40 mg, 1 time/d, then use 4 to 6 weeks; intravenous infusion of calf serum 1.6 g, once a day, for 2 weeks), surgical treatment will be performed in time when there are surgical indications. At the same time, we will pay close attention to changes in blood sugar, electrolytes and liver and kidney function to prevent and treat complications such as gastrointestinal bleeding.

#### 2.5.3 Treatment group

The treatment group will be given acupuncture treatment on the basis of the control group. We will select the following acupoints as the main acupuncture sites: Baihui, Sishenchong, Neiguan, Sanyinjiao, Xuehai, Fengchi, Quchi, Shuigou. Baihui is located at the intersection of the middle line of the head and the tip of the ears. It is closely connected with the brain and is the key point for regulating brain function. Baihui is the place where all meridians converge. Being able to reach the yin and yang veins and connect the meridians throughout the body plays an important role in regulating the balance of yin and yang in the body. Sishenchong is located 1 inch apart from the front, back, left, and right sides of Baihui on the top of the head, with a total of 4 acupuncture points. There are branches of the greater occipital nerve, ear cervical nerve and supraorbital nerve around this point. This acupuncture point mainly treats madness, epilepsy, stroke, hemiplegia, forgetfulness, insomnia, headache, dizziness, cerebral hypoplasia, hydrocephalus, head pain, and so on. Pierce 0.5–0.8 inches horizontally. The Neiguan is located 2 inches above the transverse crease of the forearm, between the palmar longus tendon and the flexor carpi radialis tendon. This point is commonly used in modern times to treat angina, myocarditis, arrhythmia, gastritis, hysteria, and so on. Pierce the skin 0.5 to 1 inch vertically. Sanyinjiao is an acupuncture point of the body. Sanyinjiao refers to the intersection of qi and blood in the 3 meridians with yin attributes on the feet. Xuehai is on the inner thigh, 2 inches above the medial end of the patellar base, and is located on the bulge of the medial head of the quadriceps when the knee is bent. Fengchi is located on the back of the neck, under the back of the skull, in the lacuna on the outer edge of the two large tendons, equivalent to the level of the earlobe. Fengchi is in the depression between the attachment between the sternocleidomastoid muscle and the upper end of the trapezius muscle. The deep layer is the splinter muscle; there are branches of occipital arteries and veins; and the minor occipital nerves are distributed. Quchi is located at the lateral end of the elbow transverse striae, at the midpoint of the line between Chize and the lateral epicondyle of the humerus when the elbow is bent. This acupuncture point has the functions of clearing away heat and removing the surface, dredging the channels and collaterals. Shuigou is on the face, at the intersection of the upper 1/3 and the middle 1/3 of the middle groove. The acupoints are distributed with the infraorbital nerve branch and the buccal branch of the facial nerve. This acupoint is mainly used for coma, syncope, heatstroke, madness, epilepsy and other diseases. Needle insertion method: puncture 0.3 to 0.5 inch diagonally upward.

Treatment time and duration. This study will use the method of moving the needle for half an hour. Acupuncture is performed once every ten minutes, once a day, 5 days a week, 10 times as a course of treatment, GCS score is recorded every 3 courses of observation, and recorded twice in a row.

### 2.6 Outcome measures

(1) There are specific indications for awakening precursors.^[4]^ We will observe the patient's specific clinical manifestations (spraying reflex and limb restlessness) during acupuncture treatment as specific indications for awakening;
(2) GCS scoring standard.^[5]^ We will perform GCS scoring on the two groups of patients before and after treatment on the 30th, 60th, and 90th day. GCS ≤ 8 points is defined as coma, GCS ≥ 9 points is defined as awakening (according to the specific clinical situation, most patients have a tracheotomy that affects the observation of the pronunciation response, so the patient's action is used as the observation scoring basis). (Table 1)

**Table 1:**
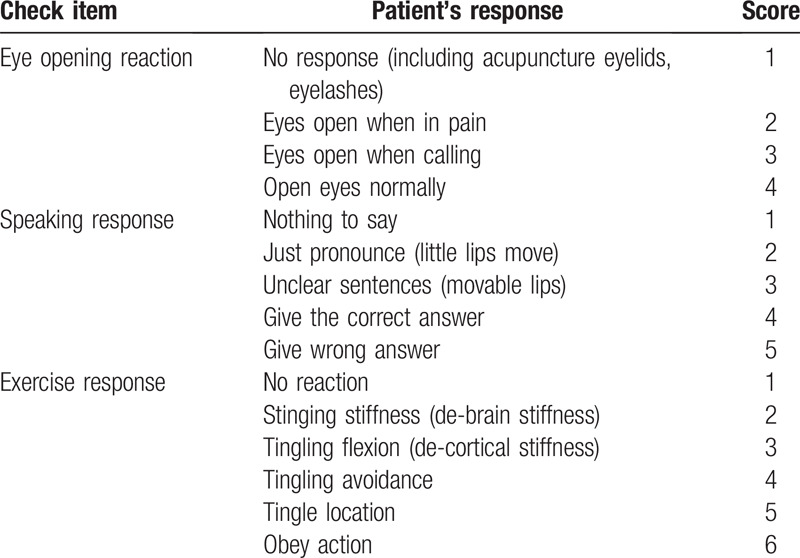
Glasgow coma scale.

Efficacy criteria. This study will use the Glasgow Outcome Scale method for the final efficacy assessment. In this study, the results will be assessed after 3 months of treatment. Grade V (good): The patient is recovering well, can live a normal life, and has mild neurological disorders; Grade IV (moderate disability): Moderately disabled, but able to take care of themselves; Grade III: Severely disabled, clear consciousness, unable to live Self-care; Level II: Plant survival; Level I: Death. Total effective rate = (good + moderate disability)/total number of cases × 100%. Glasgow Outcome Scale Grading Scale^[6]^ (Table 2).

**Table 2:**
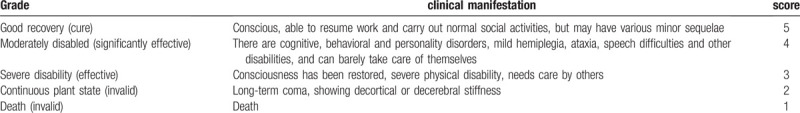
Glasgow outcome scale.

### 2.7 Data management

Information collected from participants will be stored securely in both hard copy (paper) and soft copy (electronic). The information will be stored on paper records at the Wangjing Hospital of China Academy of Chinese Medicial Sciences, and the de-identified data entered into an electronic web-based database and other research documents stored on a secure file server based at the Wang Jing Hospita of China Academy of Chinese Medicial Sciences.

All database storage is protected with login and password for individual researchers. All paper records used during the study are kept after the project has been completed for a minimum of 15 years, as per Good Clinical Practice. After the 15 years, information will be disposed of via shredding of all paper records, and deletion from databanks. Re-identifiable data is used during the conduct of the study in order to contact patients for follow-up visits. The code will be kept separately to the case report forms.

### 2.8 Data analysis

The statistical data obtained in this study will use SPSS 25.0 statistical software package for data statistics and analysis.

(1) Measurement data: expressed by “mean ± standard deviation”, t test is used for normal distribution, rank sum test is used for non-conformity, and measurement data at different time points with correlation are analyzed by repeated measurement data.
(2) Counting data; using *X*^2^ test.
(3) Rank data: use rank sum test for analysis.
(4) Results *P* < .05 means statistical difference: *P* < .01 means statistical difference is very significant.

### 2.9 Ethical

Before this clinical trial begins, we will submit the research protocol to the ethics committee for review. We will not recruit participants until the ethics committee agrees. This study will be approved by the Ethics Committee of Wang Jing Hospital of China Academy of Chinese Medical Sciences. It will be conducted in accordance with the protocol. The rules of confidentiality will be respected.

## 3 Discussion

Craniocerebral injury belongs to internal head injury in the Department of Orthopedics and Traumatology of TCM.^[7]^ TCM believes that blood stasis blocking the brain is the basic etiology and pathogenesis of brain injury. In the treatment, use the method of removing blood stasis and resuscitation. Clinical studies have shown that the treatment of promoting blood circulation and removing blood stasis can significantly improve the treatment effect of patients with severe head injury. Yin Minghua^[8]^ et al proved through animal experiments that the blood-brain barrier can pass through the blood-brain barrier, maintain the structural integrity of the blood-brain barrier during brain injury, and improve its function. And it can indirectly antagonize the elimination of free radicals and block the apoptosis of nerve cells. Die, reduce the toxicity of nitric oxide. Mo Yan^[9]^ holds that the medicine for removing blood stasis and resuscitation could be applied 48 hour after the onset. However, many scholars still believe that it is safer to start the application after the patient's condition is stable, there is no risk of rebleeding or after the acute phase (2 weeks of onset). In young people in Europe and America, the main cause of coma and disability caused by the nervous system is traumatic head injury.^[10]^ Craniocerebral injury mainly includes neuron loss of brainstem reticular structure, degeneration of myelin sheath, necrosis of ventricle or white matter nerve cells leading to focal gliosis, dural hematoma, cerebral edema and cerebral herniation caused by hyperdermal skin, etc. Pathological changes. Lesion damages the respiratory center, the patient's breathing is weakened, and the respiratory tract is easily blocked by sputum.^[11]^ Therefore, most patients with craniocerebral injury and coma need tracheotomy and sputum suction care. At present, the most widely used quantitative standards for the classification and scoring of consciousness disorder and prognosis are still the GCS and the Glasgow Outcome Scale.^[12,13]^ The diagnosis of lesions mainly relies on imaging examinations. CT and MRI have high evaluation value for lesion location and prognostic observation of coma with brain injury. Western medicine treatment programs have also taken shape. Common therapies include oxygen supply, tracheotomy, ECG monitoring, intracranial hematoma removal, reduction of internal pressure, improvement of internal blood flow, hormones, wakefulness, nutritional support, and other symptomatic treatments.^[14]^ Other awakening methods such as hyperbaric oxygen, music therapy and electrical stimulation such as deep brain stimulation (DNS), spinal cord electrical stimulation (SCS) and transcutaneous electrical stimulation have a certain awakening effect.^[15]^

With the development of social economy, transportation and various infrastructures have also developed, but it has objectively increased the number of patients with head injury. At the same time, with the continuous improvement of science and technology and medical treatment, many patients with mental disorders caused by head injury have survived through neurosurgery, medicine and professional nursing. However, the awakening treatment of long-term coma is still a problem in the world, and the long-term coma brings great damage to the financial and psychological of the patients themselves and their families, and the high coma patients also bring a huge burden to the society. Based on the above-mentioned analysis of craniocerebral injury and coma by Chinese and Western medicine, we believe that the deficiency of simple drugs should be made up. Among the non-drug treatments, acupuncture and moxibustion therapy that is safe and has little side effects has been recommended for the treatment of consciousness disorders caused by brain injury. Therefore, we designed this experiment. Our purpose is to observe the role of the method of removing blood stasis and resuscitation in promoting the recovery of patients with severe head injury.

## Acknowledgments

We will thank all patients who participated in this study. We will express our gratitude to the funding agencies of this research.

## Author contributions

**Formal analysis**: JJP and XZH.

**Investigation**: JJP and PW.

**Supervision**: WL and QZ.

**Writing – original draft:** JJP.

**Writing – review & editing**: TD.
